# Inhibition of Cardiac Kir Current (I_K1_) by Protein Kinase C Critically Depends on PKCβ and Kir2.2

**DOI:** 10.1371/journal.pone.0156181

**Published:** 2016-05-23

**Authors:** Daniel Scherer, Claudia Seyler, Panagiotis Xynogalos, Eberhard P. Scholz, Dierk Thomas, Johannes Backs, Martin Andrassy, Mirko Völkers, Christoph A. Karle, Hugo A. Katus, Edgar Zitron

**Affiliations:** 1 Department of Cardiology, Medical University Hospital Heidelberg, Heidelberg, Germany; 2 DZHK (German Centre for Cardiovascular Research), partner site Heidelberg/Mannheim, Heidelberg, Germany; Indiana University School of Medicine, UNITED STATES

## Abstract

**Background:**

Cardiac inwardly rectifying Kir current (I_K1_) mediates terminal repolarisation and is critical for the stabilization of the diastolic membrane potential. Its predominant molecular basis in mammalian ventricle is heterotetrameric assembly of Kir2.1 and Kir2.2 channel subunits. It has been shown that PKC inhibition of I_K1_ promotes focal ventricular ectopy. However, the underlying molecular mechanism has not been fully elucidated to date.

**Methods and Results:**

In the *Xenopus* oocyte expression system, we observed a pronounced PKC-induced inhibition of Kir2.2 but not Kir2.1 currents. The PKC regulation of Kir2.2 could be reproduced by an activator of conventional PKC isoforms and antagonized by pharmacological inhibition of PKCβ. In isolated ventricular cardiomyocytes (rat, mouse), pharmacological activation of conventional PKC isoforms induced a pronounced inhibition of I_K1_. The PKC effect in rat ventricular cardiomyocytes was markedly attenuated following co-application of a small molecule inhibitor of PKCβ. Underlining the critical role of PKCβ, the PKC-induced inhibition of I_K1_ was absent in homozygous PKCβ knockout-mice. After heterologous expression of Kir2.1-Kir2.2 concatemers in *Xenopus* oocytes, heteromeric Kir2.1/Kir2.2 currents were also inhibited following activation of PKC.

**Conclusion:**

We conclude that inhibition of cardiac I_K1_ by PKC critically depends on the PKCβ isoform and Kir2.2 subunits. This regulation represents a potential novel target for the antiarrhythmic therapy of focal ventricular arrhythmias.

## Introduction

Cardiac inwardly rectifying potassium current I_K1_ mediates the terminal repolarization phase of the cardiac action potential and determines diastolic membrane conductance [1]. The molecular basis of cardiac I_K1_ is formed by heterotetrameric assembly of three related channels: Kir2.1, Kir2.2 and Kir2.3. Ventricular I_K1_ has been shown to be predominantly based on heteromeric assembly of Kir2.1 and Kir2.2 channels, whereas Kir2.3 channels are more relevant on the atrial level [2–6].

In Andersen’s syndrome (Long QT syndrome 7), loss-of-function mutations in Kir2.1 are associated with reduced I_K1_ density leading to QT interval prolongation, prominent U-waves and triggered activity in the form of complex ventricular ectopy and polymorphic ventricular tachycardia [7]. Interestingly, catecholaminergic stress can trigger ventricular arrhythmia in a subset of those patients and there is considerable phenotypical overlap with catecholaminergic polymorphic ventricular tachycardia (CPVT) [7,8]. In chronic heart failure, downregulation of cardiac I_K1_ is a hallmark of the electrical remodeling process and has been shown to facilitate triggered ventricular arrhythmias [9–12].

It has been demonstrated that pathophysiologically relevant neurohumoral pathways (e.g. *via* α_1_-adrenoreceptors and ET_A_-endothelin receptors) inhibit cardiac I_K1_ through protein kinase C (PKC) dependent signalling, thereby favouring focal arrhythmogenesis by destabilisation of the resting membrane potential [13–17]. To date, twelve isoforms of PKC have been characterised and are classified based on their structure and cofactor regulation in “conventional”, “novel” and “atypical” isoforms. Targeting specific PKC isoforms for therapeutic purposes is under extensive evaluation. Kong *et al*. (2008) demonstrated that PKCβ is a crucial mediator of myocardial ischemia-reperfusion injury and hence suggested inhibition of PKCβ as a therapeutic strategy [18]. In line with this argumentation, Bowman *et al*. (1997) found that over-expression of PKCβ in the myocardium induced sudden death in neonatal mice and pathological hypertrophy in adult mice [19].

It has been recognized in various species and in human cardiomyocytes that activation of protein kinase C induces a marked inhibition of native cardiac I_K1_ [13,16,20]. Notably, it has also been shown that stimulation of α-adrenergic receptors strongly promotes focal ventricular tachycardia in a dog model of inherited arrhythmia by a decrease of I_K1_ in Purkinje fibers [15]. However, the precise molecular mechanisms underlying this regulation and the responsible cardiac PKC isoform have not been elucidated to date.

In this study, we provide evidence for the crucial role of the PKCβ isoform for the inhibition of I_K1_ by protein kinase C. We further demonstrate that Kir2.2 most likely determines PKC-sensitivity of Kir2.1/Kir2.2 heteromers, the main molecular substrate of ventricular I_K1_. This regulation may contribute to arrhythmogenesis in focal ventricular tachycardia.

## Materials and Methods

### Solutions and drug administration

Patch-clamp measurements of rat ventricular cardiomyocytes were performed in a solution containing (in mmol/l) 3.5 KCl, 140 NaCl, 1.8 CaCl_2_, 1.5 MgSO_4_, 10 HEPES and 10 glucose as described previously [21]. The pH was corrected to 7.4 with NaOH (1 mol/l). The pipette solution contained (in mmol/l) 140 KCl, 1.5 MgSO_4_, 5 KATP, 5 EGTA, 3 Na_2_-creatinphosphate and 10 HEPES. The pH was corrected to 7.4 using 1 mol/l KOH.

Patch-clamp measurements of mouse ventricular cardiomyocytes were performed in a solution containing (in mmol/l): 4 KCl, 140 NaCl, 1 CaCl_2_, 1 MgCl_2_, 0.33 NaH_2_PO_4_, 10 glucose and 10 HEPES, pH corrected to 7.4 using 1 mol/l NaOH. The pipette solution contained (in mmol/l): 140 KCl, 1 MgCl_2_, 5 KATP, 5 EGTA, 0,1 NaGTP, 3 Na_2_-creatinphosphat and 10 HEPES, pH corrected to 7.2 using 1 mol/l KOH.

For patch-clamp measurements of rat and mice ventricular I_K1_, pharmacological agents were added to the bath solution to block L-type calcium currents (10 μmol/l nisoldipine), the slow component of delayed rectifier K^+^ current (I_Ks_, 10 μmol/l chromanol), the rapid component of delayed rectifier K^+^ current (I_Kr_, 10 μmol/l E4031) and ATP-dependent K^+^ channels (K_ATP_, 1 μmol/l glibenclamide).

Two microelectrode voltage-clamp measurements of *Xenopus* oocytes were performed in a solution containing (in mmol/l) 5 KCl, 100 NaCl, 1,5 CaCl_2_, 2 MgCl_2_ and 10 HEPES as published elsewhere [22]. The pH was corrected to 7.4 with 1 mol/l NaOH. Current and voltage electrodes were filled with 3 mol/l KCl solution. Phorbol-12-myristate-13-acetate (PMA; Calbiochem), thymeleatoxin (TMTX; Calbiochem) and 3-(1-(3-Imidazol-1-ylpropyl)-1H-indol-3-yl)-4-anilino-1H-pyrrole-2,5-dione (for simplification termed “PKCβ inhibitor” in this study; Calbiochem) were dissolved in DMSO to stock solutions of 1 to 10 mmol/l and stored at -20°C. On the day of experiments, aliquots of the stock solutions were diluted to the desired concentration with the respective bath solution. The maximum concentration of DMSO in the bath did not induce any effects on the measured currents (data not shown).

### Isolation of rat and mouse cardiomyocytes

Adult rat ventricular cardiomyocytes were isolated by enzymatic dispersion by use of collagenase. In brief, rats were anesthetized with sodium pentobarbital (60 mg/kg i.p.) and heparinized and the hearts were rapidly removed and perfused in a Langendorff perfusion system. Meanwhile the heart was subsequently digested by collagenase to break down the extracellular matrix. Afterwards, the ventricles were minced and the resulting cellular digest was washed, filtered and resuspended in Medium 199 (Sigma Aldrich Chemie GmbH, Germany). Freshly isolated cardiomyocytes were attached to laminin coated cover slips by one hour incubation at 37°C and 5% CO_2_. After one hour, the medium was changed to separate the living, attached cells from the dead and further experiments were started.

Adult mice were sacrificed by decapitation. Single cells from mouse ventricular tissue were obtained using the protocol described in O´Connel *et al*. (2007) [23]. Again, the isolated cardiomyocytes were attached to laminin coated cover slips by one hour incubation and after medium change patch-clamp experiments were started.

The investigation conforms to the *Guide for the Care and Use of Laboratory Animals* published by the US National Institutes of Health (NIH publication No. 85–23, revised 1996). Approval for the animal experiments reported in this study was given by the Regierungspräsidium Karlsruhe, Germany, reference numbers G-221/12, T-78/15 and T-18/15.

### Electrophysiology and statistics

The whole-cell patch-clamp configuration was used to record currents from isolated rat and mouse ventricular cardiomyocytes. The two microlelectrode voltage-clamp configuration was used to record currents from *Xenopus laevis* oocytes as published previously [22]. All measurements were carried out at room temperature (20–22°C). Only recordings where the initial leak currents did not significantly change within the observation period were considered for data analysis. Statistical data are presented as mean ± standard error of the mean (SEM). In order to compare the statistical significance of the results, unpaired Student´s t-tests or ANOVA followed by t-test were performed: p<0.05 was considered statistically significant and p<0.01 was considered highly significant.

### Molecular biology

Complementary RNA was prepared as reported previously [22]. The human Kir2.1 clone (GenBank accession no. U12507) was a kind gift from Dr. C.A. Vandenberg (Santa Barbara, California, USA). The human Kir2.2 clone (GenBank accession no. L36069) was generously provided by Dr. B.A. Wible (Cleveland, OH, USA). The Kir2.1-Kir2.2 concatemer used in this study was generously provided by Prof. Dr. Dr. J. Daut (Marburg, Germany). Generation and characterisation of the PKCβ-knockout mice was published previously [18]. cRNA (100–600 ng/μl) was injected into stage V and VI defolliculated oocytes by using a Nanoject automatic injector (Drummond, Broomall, PA, USA). The volume of injected cRNA solution was 46 nl per oocyte. Experiments were performed 1–3 days after injection.

## Results

### Activation of PKC inhibits Kir2.2 but not Kir2.1 currents

**Fig 1:**
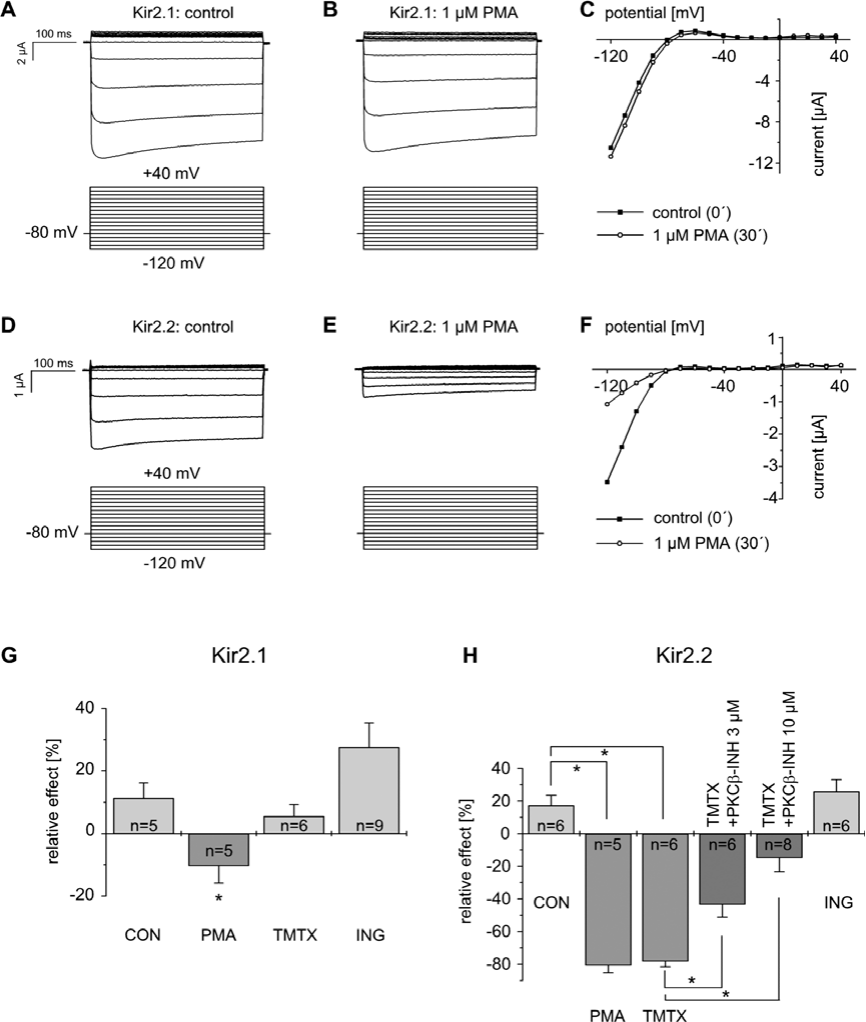
Effects of different protein kinase C activators on Kir2.1 and Kir2.2 currents indicate a pronounced inhibitory regulation of Kir2.2 by conventional PKC isoforms with a predominant role of PKCβ. Representative recordings of Kir2.1 currents before and after superfusion with PMA (1 μmol/l) for 30 minutes (A-B). Corresponding IV-curves (C). Typical recording of Kir2.2 currents under control conditions (D), after superfusion with PMA (1 μmol/l) for 30 minutes (E) and corresponding IV-curves (F). (G) Summary data of the experiments with different PKC activators is shown for Kir2.1. (H) Summary data of the experiments with different PKC activators and co-applied PKCβ inhibitor is shown for Kir2.2. * indicates statistically significant difference to control experiments. These data point to a pronounced inhibitory regulation of Kir2.2 currents by PKC that predominantly depends on conventional PKC isoforms with a crucial role of PKCβ. In contrast, the moderate inhibition of Kir2.1 channels by PMA is likely to be independent of PKC signalling pathways. Protocol: holding potential -80 mV; test pulses from -120 mV to +40 mV in 10 mV-increments (400 ms).

In order to investigate regulatory effects of PKC on homomeric Kir2.1 and Kir2.2 channels, heterologous expression of these channels was performed in *Xenopus* oocytes. A standardised voltage protocol was applied which evoked characteristic inwardly rectifying currents. Test pulses to potentials ranging from -120 mV to +40 mV were applied in 10 mV-increments for a period of 400 ms each. The holding potential was -80 mV. Steady state inward currents at -120 mV were measured to quantify effects. The observation time was 30 minutes in all experiments using *Xenopus* oocytes to ensure steady-state conditions. Fig 1A and 1D display typical recordings of Kir2.1 and Kir2.2 currents under control conditions.

Under control conditions, i.e. during superfusion with the bath solution, currents exhibited a small time-dependent run-up as reported previously [16,20]. During 30 minutes of superfusion with the bath solution, Kir2.1 currents increased to 111.3 ± 4.9% (n = 5) of the initial values. Kir2.2 channels showed a current run-up to 117.3 ± 6.1% (n = 6). Biophysical current characteristics remained unaltered (data not shown). Next, experiments were repeated with pharmacological activation of PKC by application of the phorbol ester PMA at a concentration of 1 μmol/l. This high concentration of PMA was chosen to achieve maximal PKC activation. During the observation period of 30 minutes, Kir2.1 currents showed a small but significant decrease by 10.3 ± 5.6% (n = 5; p<0.05; Fig 1B and 1G). In contrast, PMA caused a pronounced inhibition of Kir2.2 currents with a decrease by 80.7 ± 4.7% (n = 5; Fig 1E and 1H; p<0.01).

Then, we examined the effects of more specific pharmacological activators of protein kinase C with different pharmacological profiles. Thymeleatoxin (TMTX) predominantly activates conventional PKC isoforms, i.e. PKCα, PKCβ and PKCγ [24], whereas ingenol (ING) predominantly activates novel PKC isoforms, i.e. PKCδ and PKCε [25]. Notably, the presence of all known conventional as well as novel PKC isoforms in *Xenopus laevis* oocytes was shown recently [26,27]. The experiments were repeated under conditions identical to those described above, i.e. with the same voltage protocol and the same observation time. Inward currents measured before and after application of the PKC activators were compared to obtain relative effects.

For Kir2.1 channels, results are summarised in Fig 1G and for Kir2.2 channels in Fig 1H. Surprisingly, despite the moderate PMA-induced Kir2.1 inhibition, neither TMTX (100 nmol/l) nor ING (1 μmol/l) induced inhibitory effects on Kir2.1 currents. After 30 minutes of exposure currents increased to 105.5 ± 3.8% (n = 6) and 127.6 ± 7.8% (n = 9), respectively. These results were comparable to the Kir2.1 currents registered under control conditions (p>0.05). However, Kir2.1 currents also depend on PIP_2_ content in the membrane and PMA is known to activate phospholipase C [28–30]. In fact, application of a specific inhibitor of phospholipase C, D609 (100 μmol/l) [31], together with PMA (100 nmol/l) completely antagonised the PMA-induced Kir2.1 inhibition, resulting in currents comparable to those under control conditions (111.1 ± 5.5%, n = 6; p>0.05, data not shown).

In contrast, Kir2.2 currents were markedly inhibited after application of the cPKC activator TMTX (100 nmol/). Kir2.2 currents decreased to 21.9 ± 3.5% of the respective initial values (n = 6; p<0.01) which was comparable to the effect of PMA. Notably, nPKC activator ING (1 μmol/l) had no effect on Kir2.2 currents with a current increase to 125.8 ± 7.3% (n = 6) which was comparable to the measurements under control conditions (p>0.05).

In summary, these data point to a pronounced inhibitory regulation of Kir2.2 currents by protein kinase C that predominantly depends on conventional PKC isoforms. In contrast, the mild inhibition of Kir2.1 channels by PMA rather depends on phospholipase C activation without direct involvement of PKC.

### Role of PKCβ in the regulation of Kir2.2 channels

As mentioned above, conventional PKC isoforms seem to play a major role in the regulation of Kir2.2 currents. In order to identify the PKC isoform involved in the inhibitory regulation of Kir2.2 channels by PKC, we applied a small molecule inhibitor of PKCβ (3-(1-(3-Imidazol-1-ylpropyl)-1H-indol-3-yl)-4-anilino-1H-pyrrole-2,5-dione; for simplification termed “PKCβ inhibitor” subsequently) together with TMTX (100 nmol/l). As shown in the previous paragraph, application of TMTX alone at this concentration induced a pronounced Kir2.2 current inhibition (-78.1 ± 3.5%, n = 6, Fig 1H). This regulation was strongly attenuated by co-application of the PKCβ inhibitor in a concentration-dependent manner with a resulting current inhibition by 43.4 ± 7.5% (n = 6) at a concentration of 3 μmol/l and by merely 14.7 ± 8.6% (n = 8) at a concentration of 10 μmol/l (p<0.05 and p<0.01 in comparison to the effect of TMTX alone, respectively, Fig 1H). Thus, these pharmacological data support a central role of PKCβ in the regulation of Kir2.2 channels by protein kinase C.

### Pharmacological inhibition of PKCβ abolishes I_K1_ suppression by PKC in rat ventricular cardiomyocytes

**Fig 2:**
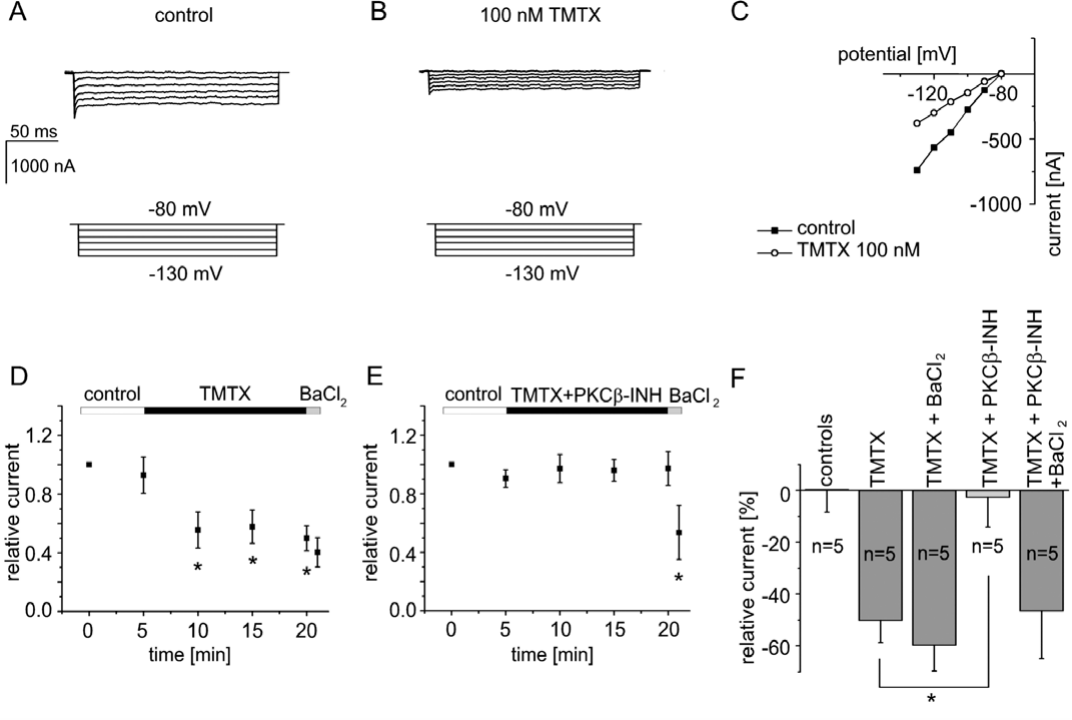
Application of a small-molecule PKCβ inhibitor abolishes the inhibitory effect of thymeleatoxin on I_K1_ in rat ventricular cardiomyocytes. Whole-cell patch-clamp experiments with isolated rat ventricular cardiomyocytes were performed using thymeleatoxin (TMTX 100 nmol/l) as PKC activator. A typical experiment is displayed in panels (A–C). Panel (D) shows the time course of effect. BaCl_2_, applied at the end of the observation period induced only a slight further current inhibition, i.e. about 84% of barium-sensitive currents had been inhibited by TMTX before. Currents remained nearly unaltered when the PKCβ inhibitor (3 μmol/l) was co-applied with TMTX (E). Subsequent application of BaCl_2_ to the bath at the end of the observation period lead to a fast block of inward currents. (F) Summary data of all experiments in rat ventricular cardiomyocytes. Protocol: holding potential -80 mV; test pulses from -130 mV to -80 mV in 10 mV-increments (200 ms). * indicates statistical significance.

In order to examine the significance of native I_K1_ regulation by PKCβ, we performed whole-cell patch-clamp experiments with isolated rat ventricular cardiomyocytes (Fig 2). The holding potential was set at -80 mV and test pulses ranging from -130 mV to -80 mV in 10 mV-increments were applied for 200 ms each. Compared to voltage-clamp experiments using *Xenopus* oocytes, the patch-clamp measurements were restricted to inward currents because of not entirely blocked sodium and calcium currents at more positive potentials. After recording a control measurement, we repeated this protocol each 60 seconds for five minutes during superfusion with the bath solution to ensure current stability. Then, TMTX (100 nmol/l) was perfused into the bath and currents were recorded in intervals of five minutes. Recordings from a typical experiment are displayed in Fig 2A–2C. I_K1_ inward currents were markedly inhibited after application of cPKC activator TMTX (Fig 2A and 2B). Summary data of the time course of effect are shown in Fig 2D. After an observation period of 15 minutes, currents were reduced to 49.9 ± 8.6% of the respective initial values (n = 5; p<0.05). At the end of each experiment, 50 μmol/l barium chloride (BaCl_2_) was applied to block residual Kir currents. Interestingly, BaCl_2_ induced only a small additional current inhibition to 40.3 ± 10% (n = 5), indicating that approximately 84% of BaCl_2_-sensitive current had already been inhibited previously by TMTX (Fig 2D). Next, we repeated these experiments using TMTX (100 nmol/l) in combination with the pharmacological PKCβ inhibitor (3 μmol/l) that was already used in the expression system as reported in the preceding paragraph. Notably, co-application of the PKCβ inhibitor almost completely abolished the effect of TMTX. After the observation period of 15 minutes, current amplitudes were almost unchanged compared to the initial values at 97.4 ± 11.5% (n = 5; p>0.05). However, the following application of BaCl_2_ to the bath induced a fast block of inward currents to 53.5 ± 18.3% (n = 5), indicating that only 6% of the BaCl_2_-sensitive current had been inhibited previously with the co-application of TMTX and PKCβ inhibitor (Fig 2E). All experiments are summarised in Fig 2F.

### Inhibitory regulation of ventricular I_K1_ is absent in PKCβ-knockout mice

**Fig 3:**
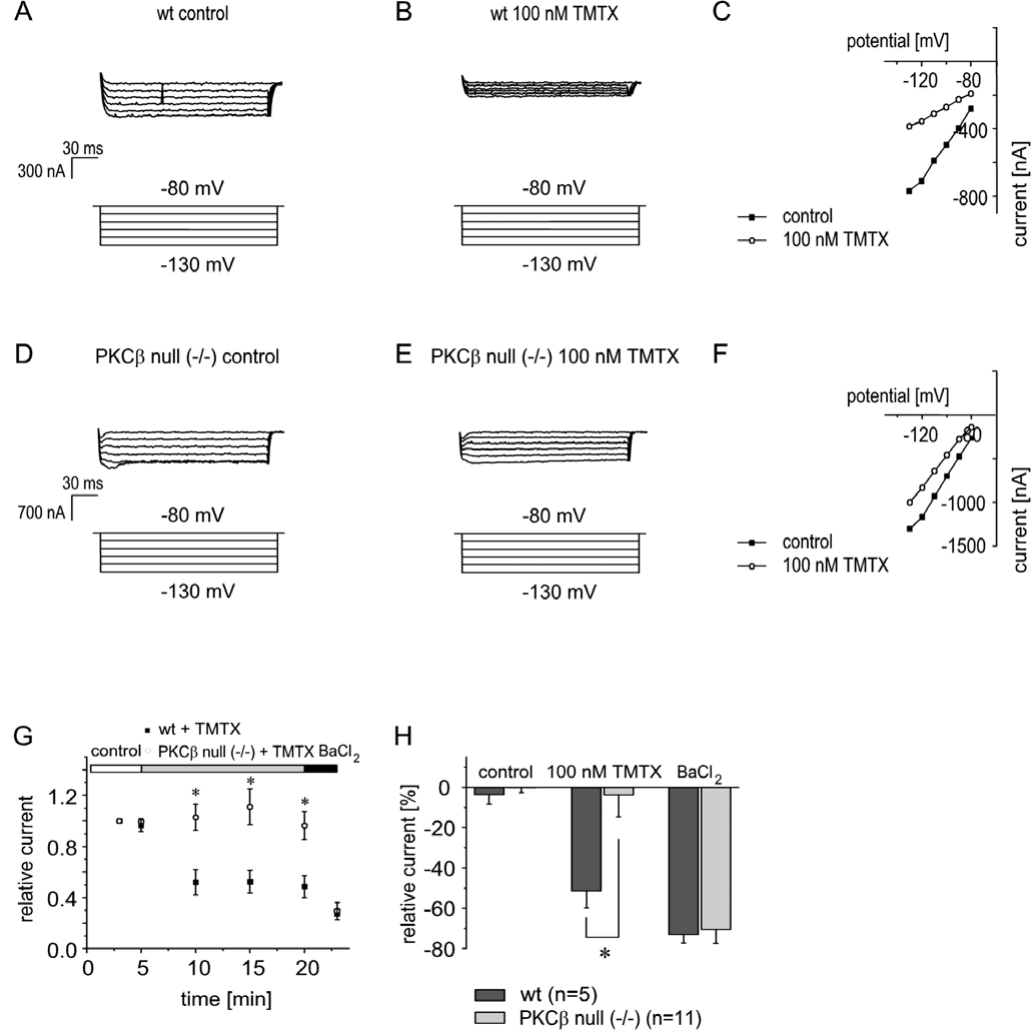
In homozygous PKCβ-knockout mice the ventricular I_K1_ is not inhibited after PKC activation with thymeleatoxin. Isolated ventricular cardiomyocytes from wild type (A-C) and PKCβ-knockout mice (D-F) were examined using the whole-cell patch-clamp configuration. Thymeleatoxin (TMTX; 100 nmol/l) was used for PKC activation. The same voltage protocol as in the experiments with rat ventricular cardiomyocytes was used. (G-H) Summary data from the experiments using cardiomyocytes from wild type and PKCβ-knockout mice. BaCl_2_ induced a rapid inhibition of inward currents in PKCβ-knockout mice but not in wild type mice, pointing to a nearly absent PKC regulation of I_K1_ in PKCβ-knockout mice. * indicates statistical significance.

In order to further validate the molecular specificity of our findings, we used homozygous PKCβ-knockout mice (PKCβ null (-/-)) [18] and wild type mice (wt) for comparative experiments. From these mice, ventricular cardiomyocytes were isolated and I_K1_ was measured as described in the previous paragraph. Initially, I_K1_ was elicited each 60 seconds for five minutes under control conditions to ensure current stability. Afterwards, cPKC activator TMTX (100 nmol/l) was applied (Fig 3A–3C). During the observation period of 15 minutes, I_K1_ from wild type mice decreased to 48.7 ± 8.6% (n = 5) of the initial values (p<0.05). The subsequent application of BaCl_2_ (50 μmol/l) only induced an additional reduction of inward currents to final amplitudes of 27 ± 4.2% (n = 5) of the initial values, demonstrating that approximately 70% of BaCl_2_-sensitive current had been inhibited by the preceding application of TMTX (Fig 3G and 3H).

Ventricular cardiomyocytes from PKCβ-knockout mice were subjected to the same protocol (Fig 3D–3F). Interestingly, in those cells the effect of TMTX was almost completely abolished. After 15 minutes of wash-in of TMTX (100 nmol/l), Kir current amplitudes remained at 96.3 ± 11% (n = 11) of the respective initial values (p<0.01 in comparison to the effect of TMTX in wild type cardiomyocytes). The subsequent application of BaCl_2_ into the bath to block residual inward currents induced a rapid reduction of inward currents to 29.4 ± 6.7% of the respective initial values (Fig 3G and 3H) indicating that in cardiomyocytes from PKCβ-knockout mice, TMTX inhibited only 5% of the barium-sensitive current.

### Heteromeric Kir2.1/2.2 channels are inhibited after activation of PKC

**Fig 4:**
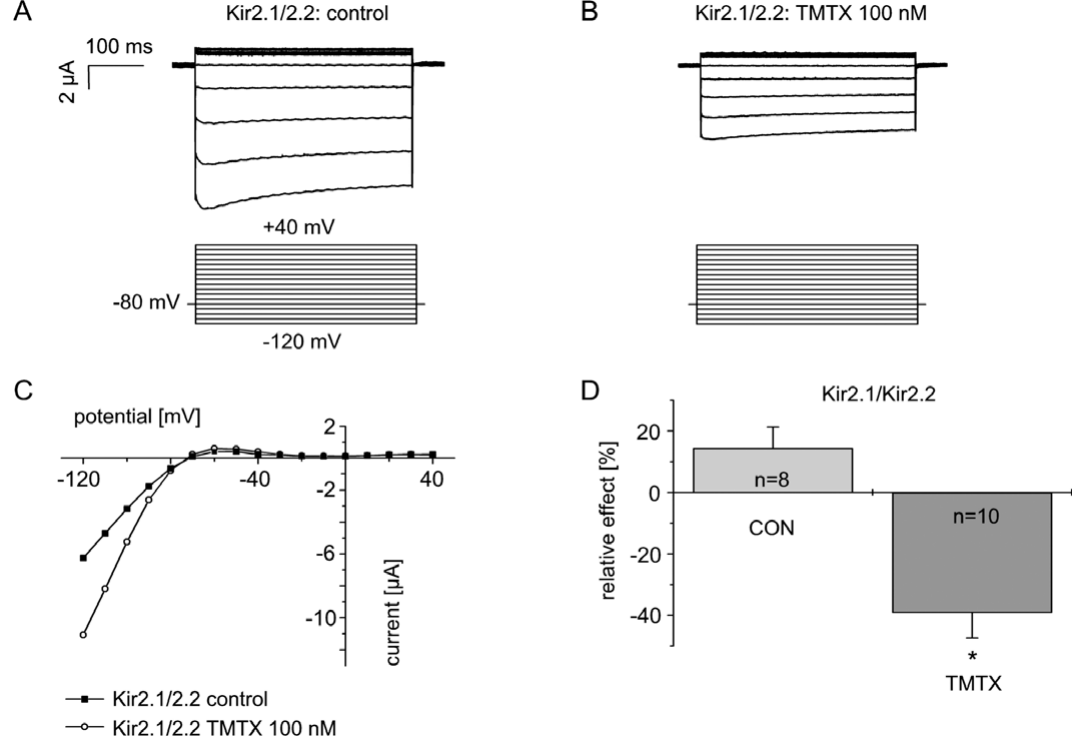
Heteromeric Kir2.1/Kir2.2 channels are inhibited by protein kinase C. Heteromeric Kir2.1/Kir2.2 channels were expressed as concatemers according to Preisig-Müller *et al*. (2002) [3]. (A-C) A representative experiment and corresponding IV-curves. (D) Summary data of relative currents upon activation of PKC by thymeleatoxin (TMTX; 100 nmol/l) compared to measurements of Kir2.1/Kir2.2 heteromeric channels under control conditions. * indicates statistical significance.

It has been reported that Kir2.1 and Kir2.2 are the predominant Kir2.x subunits in human cardiac ventricle, and that heteromeric assembly of Kir2.1 and Kir2.2 is likely to be the main molecular correlate of human ventricular I_K1_ [4]. Furthermore, it has been shown that co-expression of Kir2.1 and Kir2.2 in *Xenopus* oocytes gives rise to currents with distinct biophysical properties that resemble those of human native I_K1_ better than those of homomeric Kir2.1 or Kir2.2 currents [3,4]. Based on the presented data showing that Kir2.2 subunits are inhibited by protein kinase C whereas Kir2.1 subunits are not, we were interested in investigating the PKC regulation of Kir2.1/Kir2.2 heteromers. Preisig-Müller and co-workers (2002) expressed heteromeric Kir2.1/Kir2.2 channels generated by concatenating the channels´ RNA and expressing them as a single RNA chain resulting in “concatemers” [3]. We also used this approach to express heteromeric Kir2.1/Kir2.2 channels in *Xenopus* oocytes. Kir2.1/Kir2.2 heteromeric currents were measured and effects were quantified as described for homomeric channels above. Typical currents after heterologous expression of Kir2.1-Kir2.2 concatemers in *Xenopus* oocytes under control conditions and after application of TMTX (100 nmol/l) and the respective current-voltage relationship are shown in Fig 4A–4C. Under control conditions, heteromeric Kir2.1/Kir2.2 currents increased to 114.4 ± 6.9% (n = 8) which is comparable to the previously described run-up of the homomeric Kir2.1 and Kir2.2 channels. After exposure to TMTX, currents decreased to 61 ± 8.3% (n = 10, p<0.01, Fig 4D). In view of the fact that Kir2.1 is insensitive to regulation by PKC as shown above, we therefore propose that co-heteromerization of Kir2.2 subunits confers PKC-sensitivity to the Kir2.1/Kir2.2 heterotetramers.

## Discussion

Although the inhibitory regulation of cardiac I_K1_ by the protein kinase C system has been demonstrated to be of major pathophysiological relevance for focal arrhythmogenesis, the underlying molecular mechanisms have only partially been elucidated to date [13–17]. To the best of our knowledge, this is the first study providing comparative data on the regulation of homomeric and heteromeric Kir2.1 and Kir2.2 channels and native I_K1_ by protein kinase C isoforms. Our data demonstrate that protein kinase C signalling induces a pronounced inhibition of homomeric Kir2.2 channels but not of Kir2.1 channels. This regulation is predominantly mediated by conventional PKC isoforms with a crucial role of the PKCβ isoform as demonstrated in cardiomyocytes from PKCβ-knockout mice. Kir2.2 channel subunits most likely mediate PKC-sensitivity of Kir2.1/Kir2.2 heteromers.

### Differential regulation of homomeric Kir2.1 and Kir2.2 channels by PKC

We observed a pronounced inhibition of Kir2.2 channels by PKC while Kir2.1 channels were insensitive to this regulation. In *Xenopus* oocytes, Kir2.2 currents showed a marked inhibition after application of the phorbol ester PMA that is commonly used as a potent activator of PKC. However, also the more specific PKC activator thymeleatoxin induced an inhibition of Kir2.2 currents. In contrast, Kir2.1 currents showed only a mild inhibition by PMA but neither by thymeleatoxin nor ingenol. However, the PMA-induced Kir2.1 inhibition was abolished after co-application of D609, a specific phospholipase C inhibitor. Therefore, we propose that a PKC-sensitivity of Kir2.1 channels is unlikely and that the effect of PMA is probably caused by an activation of phospholipase C with a subsequent reduction of the membrane PIP_2_ content independent of PKC [28,29].

Different studies of the modulation of Kir2.1 channel activity by PKC have been performed with contradicting results. Initially, Fakler and co-workers (1994) observed a Kir2.1 channel inhibition by PKC which was later questioned due to the non-physiological experimental setting in excised inside-out patches [32]. Accordingly, in whole cell-experiments two other groups did not observe regulatory effects of PKC on homomeric Kir2.1 channels [33,34].

**Fig 5:**
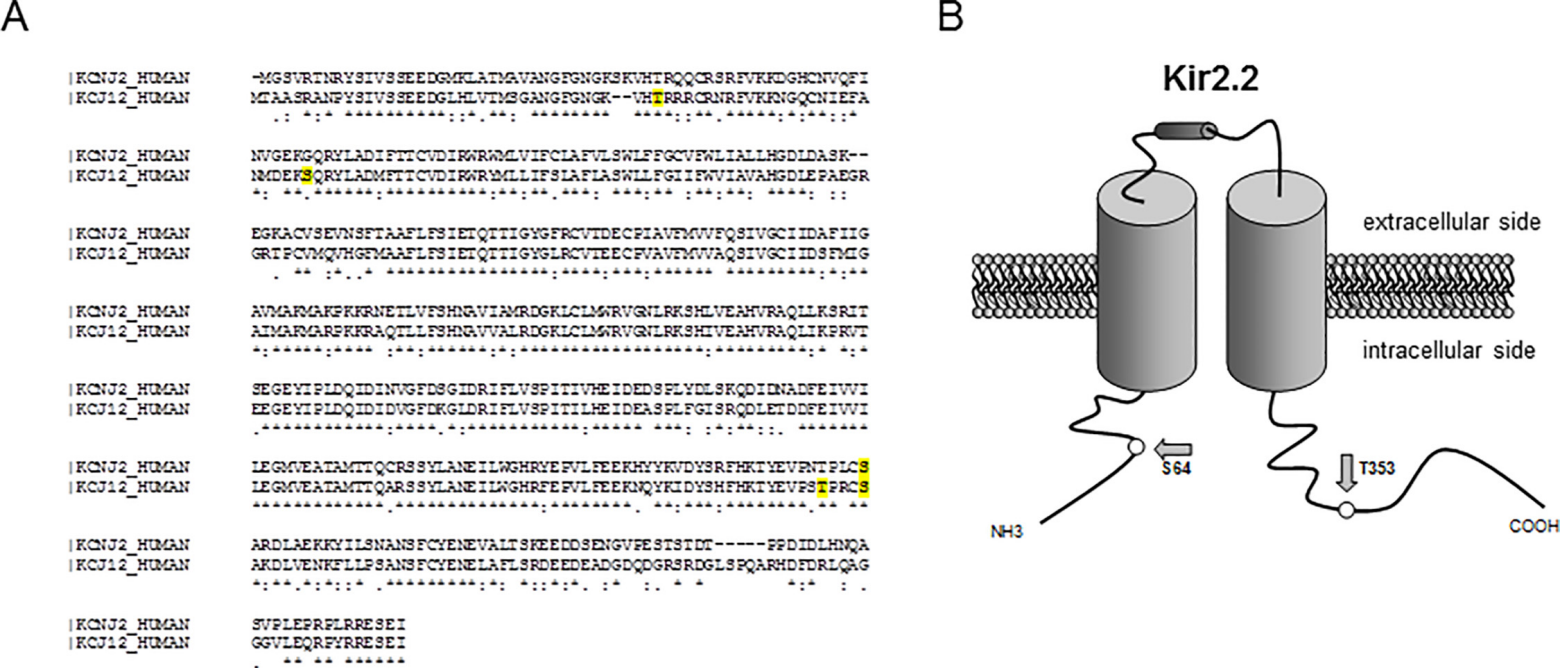
The functional PKC consensus sites S64 and T353 are lacking in Kir2.1 channels. (A) Alignment of the amino acid sequence of Kir2.1 and Kir2.2. Interestingly, in Kir2.1 channels, the PKC consensus sites S64 and T353 which have been shown to be functionally relevant are lacking. (B) Schematic picture of a Kir2.2 channel subunit with the suggested locations of the PKC phosphorylation sites.

In an earlier work from our group, using the computer program HUSAR Prosite (DKFZ, Heidelberg, Germany) to search for key sequences for PKC phosphorylation (amino acids S/T-X-R/K) in Kir2.2 channels, 4 putative sites (T38, S64, T353, S357) were identified with S64 and T353 have been emerged to be of functional relevance [20]. We performed an alignment between human Kir2.1 and Kir2.2 channel sequences (Clustal Omega software, European Molecular Biology Laboratory—European Bioinformatics Institute, Cambridge, UK), demonstrating that the PKC consensus sites T38, S64 and T353 are lacking in Kir2.1 channels (Fig 5A and 5B). Unless the lack of these consensus sites provides a plausible mechanism why Kir2.1 channels are insensitive towards PKC regulation we did not show direct phosphorylation of PKC at Kir2.2 residues and therefore other mechanisms, leading to Kir2.2 channel inhibition downstream PKC signalling are not fully excluded.

### Inhibition of ventricular I_K1_ depends on conventional PKC isoforms with an essential role of PKCβ

It has been demonstrated previously that ventricular I_K1_ is inhibited by protein kinase C dependent signalling [13,15,16]. However, the underlying molecular mechanisms had not been examined in further detail in the respective studies. Based on the pharmacological data obtained in the expression system we hypothesized that this regulation is mediated by conventional PKC isoforms with a central role of PKCβ. In line with this notion, we found in rat ventricular cardiomyocytes that cPKC activator thymeleatoxin induced a pronounced inhibition of I_K1_ and that this effect was suppressed by co-application of a small-molecule PKCβ inhibitor. Finally, we obtained ventricular cardiomyocytes from PKCβ-knockout mice to test our hypothesis at a level of molecular specificity superior to pharmacological approaches. Supporting our hypothesis, we observed a marked inhibitory regulation of ventricular I_K1_ by protein kinase C in wild type cardiomyocytes that was completely absent in cardiomyocytes from PKCβ-knockout mice indicating that PKCβ is essential for this regulation.

### Kir2.2 channels mediate PKC-sensitivity of heteromeric Kir2.1/Kir2.2 channels

The main molecular correlate of ventricular I_K1_ is heterotetrameric assembly of Kir2.x channels with Kir2.1 and Kir2.2 being the predominant subunits [3,6]. As Kir2.2 showed a pronounced regulation by PKC pathways but Kir2.1 channels did not, we were interested to study the PKC regulation of heteromeric channels. We used Kir2.1–2.2 concatemers provided by Preisig-Müller and co-workers (2002) to generate heteromeric channels [3]. Expression of these concatemers results in heterotetrameric channels consisting of two Kir2.1 and Kir2.2 subunits, respectively. Kir2.1/Kir2.2 heteromers exhibited a pronounced PKC-sensitivity in spite of the fact that homomeric Kir2.2 channels were lacking in the membrane. Hence, we propose that the presence of Kir2.2 channel subunits *within* a heteromer determines its sensitivity to PKC regulation.

### Potential implications on cellular electrophysiology

In cardiomyocytes, proarrhythmic signalling through adrenergic α_1A_ receptors, angiotensin receptors and endothelin receptors is associated with PKC translocation, leading to a subsequent inhibition of I_K1_ [13,15,16]. The subsequent destabilization of the resting membrane potential strongly facilitates the generation of delayed afterdepolarisations, triggered activity and ventricular tachyarrhythmias [7,12]. Hence, the PKCβ dependent inhibition of I_K1_ may be an antiarrhythmic target in the therapy of focal ventricular tachycardia.

## Conclusion

In this study, we provide evidence for the regulation of cardiac I_K1_ by conventional protein kinase C isoforms with a central role of the PKCβ isoform. As Kir2.1 channels are insensitive to PKC regulation, the regulation of heteromeric channels by PKC depends on Kir2.2 subunits. Due to its proarrhythmic potential, this regulation represents a potential target for the therapy of focal ventricular tachycardia.
